# Life threatening pancreatitis with panniculitis: A sequelae of weight gain supplement

**DOI:** 10.12669/pjms.41.5.10072

**Published:** 2025-05

**Authors:** Aneela Hussain, Afza Naureen Ghouse, Afia Shafiq

**Affiliations:** 1 Aneela Hussain, FCPS (Internal Medicine), FCPS (Infectious Diseases) Consultant Infectious Diseases, The Indus Hospital, Karachi, Pakistan; 2 Afza Naureen Ghouse, FCPS(Dermatology) Consultant Dermatology, The Indus Hospital, Karachi, Pakistan; 3 Afia Shafiq, FCPS (Histopatology) The Indus Hospital, Karachi, Pakistan

## Abstract

Panniculitis is a rare dermatological condition that can be caused by numerous factors. Pancreatic pathologies including acute pancreatitis is one of the rarer causes of panniculitis. Panniculitis heals when the etiological agent is removed. This means that in the case of Pancreatic panniculitis, panniculitis begins healing when the pancreatitis settles. Anabolic steroid containing mass-gainers used in body building can cause acute pancreatitis as stated in literature. The association of mass-gainer associated acute pancreatitis leading to panniculitis is an interesting feature rarely reported. In this case report we present a young man who developed a devastating Pancreatic Panniculitis after mass-gaining supplement intake.The case was diagnosed on characteristic histopathology findings.

## INTRODUCTION

Pancreatic panniculitis is a rare skin manifestation secondary to acute or chronic pancreatitis. It was first reported by *Hans Chiari* as far back as in the year1883.^1^ However, this kind of panniculitis was not formally documented in literature until 1947 when Szymanski and Bluefarb identified it as a feature of pancreatic pathology.^2^ It presents as reddish brown nodules which ulcerate and later develop atrophic changes.^3^ These lesions can be difficult to differentiate from other chronic skin conditions like nodular vasculitis, sarcoidosis, polyarteritis nodosa, erythema nodosum, and cutaneous abscess to name a few.^4^,^5^ As few as 2-3% of cases of panniculitis occur in conjunction with pancreatitis.^6^ Panniculitis is marked by subcutaneous inflammation due to a variety of causes including infection, and autoimmune conditions.^3^ Drug-induced pancreatitis is an uncommon condition where mostly Azathioprine and tyrosine kinase inhibitors are reported. Steroids are considered a less notable cause in comparison.^3^,^7^ Having said that, steroids are a recognized cause of Pancreatitis.^8^ Mass-gaining supplements causing pancreatitis is an even less heard of an association and there was a lack of any information on this in literature. In this instance, we report a case of pancreatic panniculitis brought on by a weight-gain supplement believed to contain steroids.

## CASE PRESENTATION

A 32 years old, unmarried man, presented to the ER with a two-month history of bullous lesions on lower limbs and fever, low grade, and a couple of days history of oral ulcers and abdominal pain. The bullous lesions were of variable size, starting on the dorsum of the right foot and increasing to a maximum of 5x5 cm, they usually burst to release a blood-tinged serous to yellowish pussy discharge with a remnant of an ulcerated plaque-like lesion which he bandaged himself. There were around six such lesions bilaterally on his calves, thighs, and feet. (Fig.1 and 2).

Apart from these, there were small, painful, pea-sized, circular, reddish ulcers in the buccal cavity with overlying white patches, easily scrapped off, and angular cheilosis causing much difficulty in eating. There was acne on his face and shoulders, bruises on his arms and yellow, thick nails which had all happened over the last two months. He had other symptoms of difficulty walking due to lethargy despite a normal neurological examination. He had taken broad-spectrum antibiotics, like Ceftriaxone and then Augmentin, on and off during this time with no help.

**Figure:**
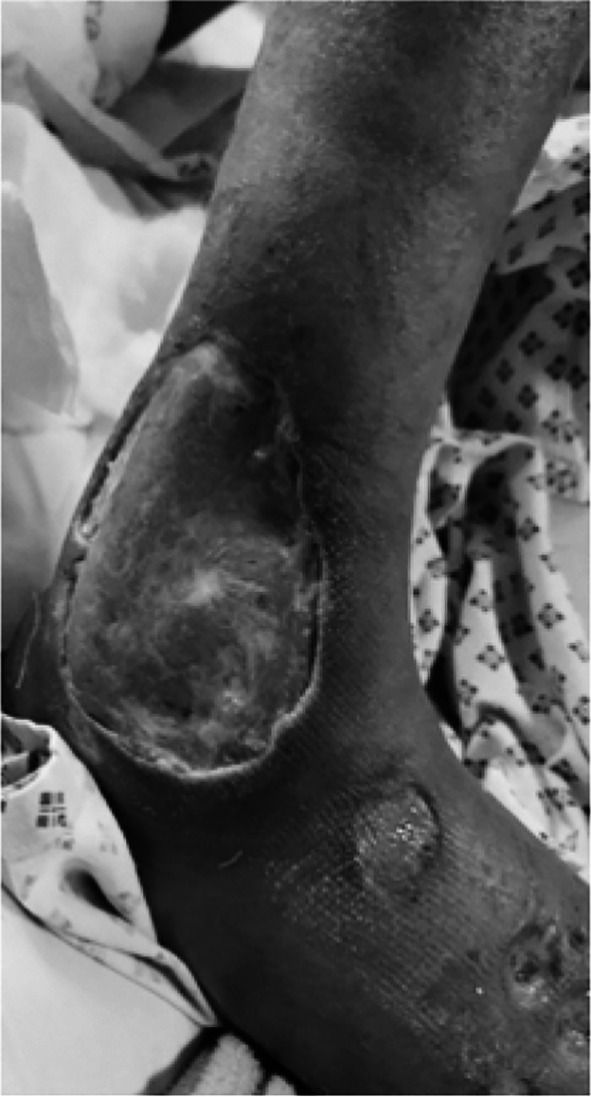
Fig.1

**Figure:**
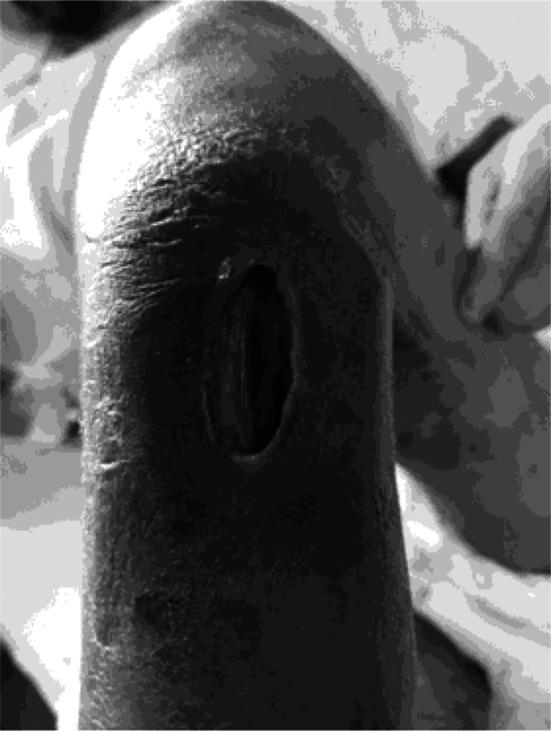
Fig.2

There was a three-month history of taking some weight-gaining supplement over the internet from an unregistered source which he blended in water in the form of two scoops a day. No ingredients were mentioned on the packaging and the sample was not available for us to analyze. He had stopped taking this a month ago as the online site had gone out of reach. He noticed putting on a weight of eight kilograms, mainly around his trunk and face giving him a cushingoid appearance

During admission his blood pressures were in the range of 118-120/60-65 mm of Hg, pulse was 90-110/minute with a normal respiratory rate, and daily fevers of 101-102 ^o^F, and the following laboratory workup was received. Hemoglobin 7.8 g/dl, mean corpuscular volume 80 fl, Total leukocyte count 11,000 mm^3^, platelets 150,000 mcL, Serum Creatinine 2.49 mg/dl, Urea 60 mg/dl, C- Reactive Protein 2.49 mg/L, ESR 119 mm/hr Liver function tests were normal, Serum electrolytes showed a Sodium of 128 mEq/l, Potassium 3.8 mEq/l, chloride 106 mEq/l Bicarbonate 14.6 mEq/l, HIV, HBsAg and Anti HCV were all negative, Total protein Albumin to Globulin ratio 0.79, Serum Calcium 7.7 mg/dl, Vitamin D 12.8 ng/ml, Serum Albumin 2.3 g/dl, LDH 934 U/l, Random blood sugars were within normal range, Serum cortisol at midnight 11.3 micg/dl, 24 hour Urinary cortisol 47.6 micg/24 hours, ANA and DsDNA were negative. His amylase and lipase were initially 75 U/L and 38 U/L respectively, however on repeat testing the levels rose up to 449 U/L and 618 U/L respectively.

He was started on Clindamycin and orthopedic surgery services were taken for debridement of the leg wounds. The culture taken grew Extended Spectrum Beta-lactamase producing Escherichia Coli. And Clindamycin was switched to Meropenem. Echocardiography showed an Ejection fraction of 33%, moderate global Left ventricular systolic dysfunction, mildly dilated aortic root with mild to moderate Aortic regurgitation, mild Mitral regurgitation, and a Grade-1 left ventricular diastolic dysfunction. CT Abdomen showed a Right Iliac fossa pseudocyst measuring 7x7 cm and a swollen pancreas withextensive inflammation and 40% necrosis. An ultra-sound guideddrain was placed in the Right inguinal fossa pseudocyst which later grew Carbapenem-Resistant Klebsiella pneumoniae resistant to all antibiotics except Tigecycline. Histopathology report of the leg lesions showed dense acute on chronic lobular pattern of inflammation in fat lobules consistent with panniculitis. (Fig.3 and 4) On meropenem, the patient became afebrile within 72 hours and so that was continued for 14 days. His leg lesions also started improving and some completely healed.

**Figure:**
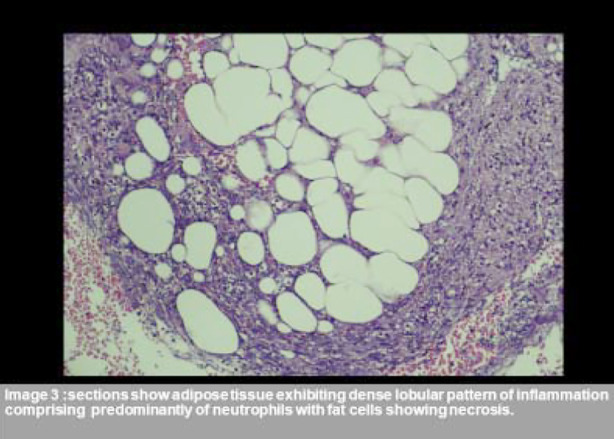
Fig.3

**Figure:**
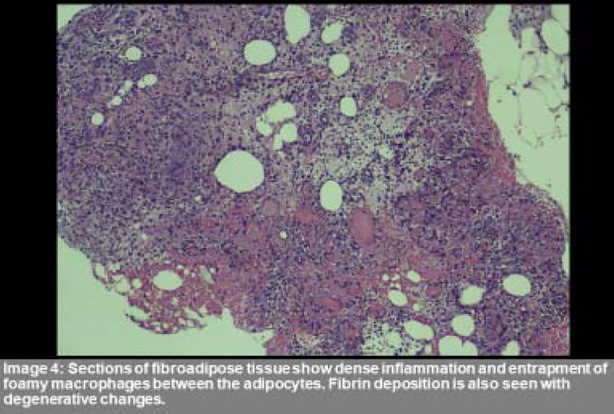
Fig.4

The patient, however, developed new fevers during admission and went into septic shock and ARDs secondary to Intravenous line sepsis from Carbapenem Resistant Pseudomonas and Candida Glabrata and despite appropriate antibiotics could not be revived. We later found out that two of his friends had also used this supplement. They were admitted elsewhere and met a similar fate.

## DISCUSSION

Numerous factors can lead to acute pancreatitis, such as autoimmune diseases, trauma, toxins, hypertriglyceridemia, hypercalcemia, cholelithiasis, persistent alcoholism, and infections. Steroids, thiazides, vinca alkaloids, didanosine, azathioprine, and other medications are also contributing causes.^8^ Pancreatic Panniculitis is seen in Pancreatitis, panniculitis, and polyarthritis syndrome (PPP) mostly, however, our patient did not show any signs or symptoms of arthritis, which when present is usually migratory.^4^,^5^ Another interesting finding is that panniculitis associated with pancreatitis involves the lower limbs 98% of the time and our patient had all his lesions on the legs.^5^ Most cases are seen in men, almost four times as many, and in the 40-60- age group which contrasts with our patient who was in his early thirties.^4^,^5^

Benign pancreatic diseases such as acute or chronic pancreatitis are more common than pancreatic cancer as causes of pancreatic panniculitis.^9^ Acute pancreatitis has been studied as the most common condition of the pancreas causing this skin lesion with a reported mortality of 27%.^5^ Some patients may be asymptomatic, therefore, in the presence of panniculitis, a CT-imaging should be ordered to avoid diagnostic delay.^4^,^7^

Mostly, the causative agent for pancreatitis has been gallstones or alcohol use. Only a handful of cases of steroid induced pancreatitis have been reported.^4^,^10^ A case of pancreatitis linked to the anabolic steroid trenbolone acetate for improving sports performance was described by Kumar et al.^11^ Other cases are post-ERCP, traumatic pancreatitis, pancreatic cancers, and pancreas divisum.^4^ Interestingly, a case of Azathioprine related PPP syndrome has been reported after six days of ingestion.^7^ Young people and athletes frequently use anabolic steroids to gain more muscle mass and, eventually, improve their physical performance. According to a local survey, anabolic steroids were utilized by 60.8% of the male participants who attended the gym.^12^ Our case is unique in the sense that the patient developed pancreatitis after using a weight-gainer, which also led to his cushingoid appearance . This may signify the presence of steroids in the supplements. Having said that, the patient did not have any samples for us to test our assumption. . Another interesting feature is that his panniculitis developed around three months after stopping the supplement which is another interesting feature that may have impacted his hormone tests.

The differential diagnosis of panniculitis includes Sarcoidosis, Behcet’s disease, and Polyarteritis nodosa therefore, histopathological confirmation is a requirement.^5^ The pathogenesis of panniculitis is related to lipase leaking out to the peripheral tissues and causing lipolysis, fat necrosis, and inflammation leading to red nodules, which subsequently ulcerate to release a brownish fluid.^4^,^5^,^7^ Similar pathology is seen in polyarthritis where the pancreatic enzymes affect the synovium.^4^ The characteristic finding on histopathology are “ghost cells”, with lobular fat necrosis and neutrophilic infiltration.^4^

Though the treatment is usually supportive, panniculitis mostly mirrors the healing of the pancreas.^4^ There has never been a report in the literature of pancreatitis caused by steroids coupled with panniculitis. Given that two of his friends had a similar presentation and a comparable history of using weight-gaining supplements, the diagnosis in our study is strongly indicative of steroid usage.

This case is reported to delineate the diagnosis of pancreatic panniculitis through proper imaging, blood tests and biopsy. We have not found any other case of weight-gain supplements leading to pancreatic panniculitis and this adds to the literature as a novel finding.

### Author`s Contribution:

**AH:** Primary author, manuscript writing, reviewing, data collection and literature search.

**ANG:** Literature search, manuscript writing and review.

**AS:** Data collection and review.

All authors have read, approved the final version and are accountale for the integrity of the study.
